# *N*-Glycomics of Cerebrospinal Fluid: Method Comparison

**DOI:** 10.3390/molecules26061712

**Published:** 2021-03-19

**Authors:** Byeong Gwan Cho, Cristian D. Gutierrez Reyes, Yehia Mechref

**Affiliations:** Department of Chemistry and Biochemistry, Texas Tech University, Lubbock, TX 79409, USA; Andrew.cho@ttu.edu (B.G.C.); Cristian.D.Gutierrez-Reyes@ttu.edu (C.D.G.R.)

**Keywords:** glycomics, cerebrospinal fluid, permethylation, LC-MS

## Abstract

Cerebrospinal fluid (CSF) contains valuable biological and neurological information. However, its glycomics analysis is hampered due to the low amount of protein in the biofluid, as has been demonstrated by other glycomics studies using a substantial amount of CSF. In this work, we investigated different *N*-glycan sample preparation approaches to develop a more sensitive method. These methods, one with an increased amount of buffer solution during the *N*-glycan release step with a lower amount of sample volume and the other with Filter-Aided *N*-Glycan Separation (FANGS), were compared with recent work to demonstrate their effectiveness. It was demonstrated that an increased amount of buffer solution showed higher intensity in comparison to the previously published method and FANGS. This suggested that digestion efficiency during the *N*-glycan release step was not in an optimal condition from the previously published method, and that there is a substantial loss of sample with FANGS when preparing *N*-glycans from CSF.

## 1. Introduction

In the last years, omics science have become the major field of study in cellular and molecular systems providing a better understanding of human disease [1]. Each type of omics provides a list of differences associated with disease stages, which is information that can be useful to evaluate its progression and for marker development. Additionally, the integration of multiple omics provide information of the original cause of disease, functional consequences, or relevant interactions [2]. Genomics [3], transcriptomics [4], proteomics [5], glycoproteomics [6], metabolomics [7], lipidomics [8], and glycomics [9] are some of the omics analysis more often investigated by the researchers in the field. Recently, glycomic profiles of different human fluids such as blood, urine, saliva, and cerebrospinal fluid (CSF) have been associated with the progression of multiple diseases [10,11,12,13,14]. Both serum and plasma are the fluids of choice to perform glycomic analysis since unlike CSF, blood samples are easy to acquire. Otherwise, CSF has become a main source of neurochemical information for cognitive disorders. Serum and plasma fluids transport molecular information between the brain and the periphery, which make them target fluids for diagnostic purposes. Although these fluids are easily accessible, they also have important disadvantages. The serum and plasma proteome have several orders of magnitude in concentration, and the changes in protein abundance must exceed the normal range to be considered biologically relevant. Thus, these samples must be subjected to exhaustive separation processes such as depletion, which separates high and low abundant proteins, or specific protein purification methodologies using antibodies targeting known disease-related glycoproteins [15]. Unlike the other above-mentioned human biofluids, CSF need a more invasive extraction procedure. Its direct contact with the brain and spinal cord makes this fluid a reliable analytical sample for the identification of biochemical changes that occur during the progression of neurodegenerative diseases [10,16,17]. Currently, CSF is a source of biomarkers for diagnostic workups of Alzheimer’s disease (AD), mild cognitive impairment (MCI), multiple sclerosis (MS), Parkinson’s, and other related diseases [18,19,20,21]. However, it has long been known that in early, presenile, and atypical cases, and in the presence of comorbidities, the diagnostic accuracy of current biomarkers may drop substantially [16].

Among the major changes observed in CSF when a disease develops, alterations in the protein glycosylation process generate differences in the glycome that in many cases can be directly related with a disease stage (e.g., change in abundance of a glycan or group of glycans, or the modification of the sialic acid linkage in sialylated glycans). A number of neurodegenerative studies based on CSF glycomics have described significant changes in abundances for the bisecting *N*-acetylglucosamine (GlcNAc) and core fucose glycans, which are structures known as “brain type *N*-glycans” [22,23,24,25,26,27,28]. Thus, glycans are valuable biomolecules that may serve as potential biomarkers [10,22,29]. Although CSF is a useful source of information for neurodegenerative diseases, its complex matrix and low glycoprotein concentration present arduous challenges in glycomic studies [30,31]. In the past, the comprehensive characterization of the CSF glycome has required several hundred microliters of fluid [22,32] and highly sensitive LC-MS analytical strategies. Additionally, glycan analysis using reverse-phase liquid chromatography and mass spectrometry (RPLC-MS) should be accompanied by a derivatization technique to overcome the poor retention of native glycans in common reverse phase (RP) columns, as well as their low ionization efficiency in positive mode [33].

In this work, we present a sensitive CSF glycan analytical strategy that used as little as 15 μL of starting material; as far as we know, there is no other analytical application using such a small sample volume with reproducible results. The sample preparation was complemented with a SPE-C18 (solid phase extraction) glycan purification to avoid undesirable matrix components [34]. This was followed by permethylation that enhanced the glycan hydrophobicity, thereby increasing ionization efficiency and rendering them amenable to positive ionization. Permethylated glycans were also well retained in RP columns where their separation facilitated avoiding competitive ionization and simplified spectral interpretation [33]. This strategy was directly compared against the benchmark method (Cho et al. [10]) using 50 μL of CSF and Filter-Aided *N*-Glycan Separation (FANGS) [35,36]. We also found that preparing glycan samples using FANGS protocol showed distorted distributions of glycans when compared to in-solution methods. Despite the reduction of the starting sample and injection volume, 5 µL, our strategy produced higher peak intensities than the benchmark and FANGS methods, which allowed us to perform more sensitive analysis of the CSF *N*-glycome.

## 2. Results

**Figure 1:**
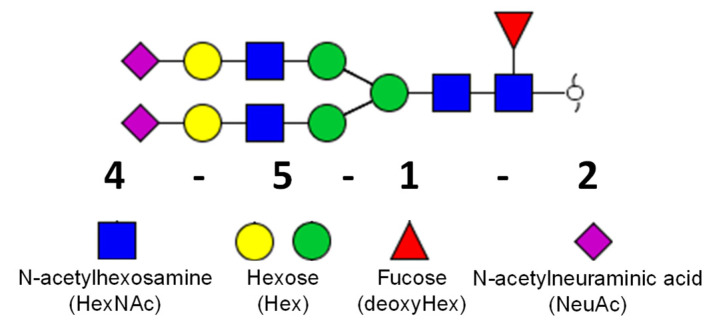
Nomenclature of glycan structure used in this study. Glycan depicted above represents the composition of four HexNAc, five Hex, one deoxyHex, and one NeuAc (4-5-1-2). The green circle represents mannose and the yellow circle represents galactose.

The nomenclature of glycan structures is described in Figure 1.

**Figure 2:**
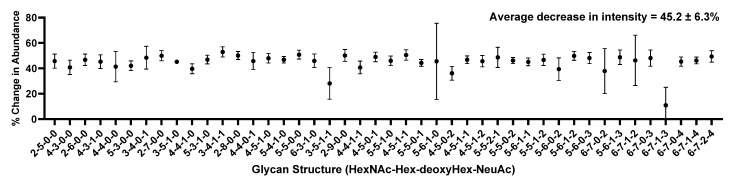
Scatter plot showing decrease in instrument signal as a result of reducing the injection amount from 10 µL of cerebrospinal fluid (CSF) to 5 µL of CSF. As expected, the signal was reduced by nearly half.

Initially, the attempt to reduce the injection amount of CSF required to perform *N*-glycome profiling was investigated using the previously published method by Cho et al. [10] as a benchmark, where *N*-glycans were prepared from 50 μL of CSF, but the injection amount was equivalent to 10 μL of CSF. In this study, the injection amount was halved to assess the possibility of performing *N*-glycan profiling of CSF with less material. Figure 2 depicts the result of reducing the injection amount by half.

**Figure 3:**
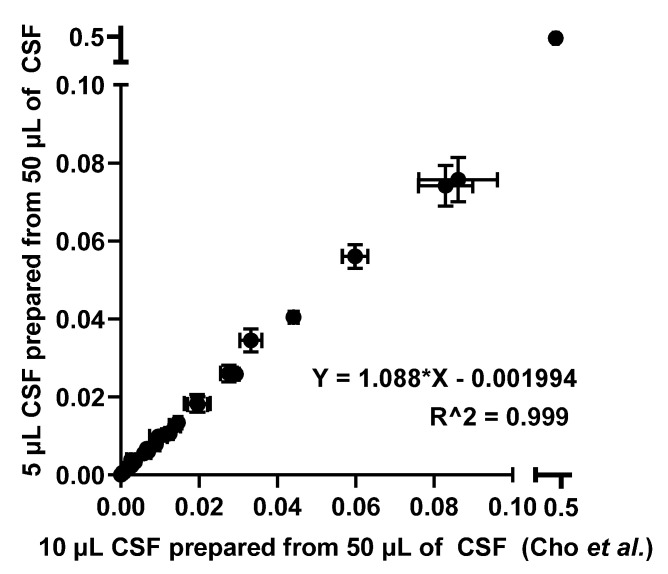
Scatter plot describing a relative quantitation comparison between the two different injection amounts.

Predictably, reducing the injection amount to 5 µL resulted in a decrease of the signal by nearly half. A relative quantitation comparison of *N*-glycans derived from CSF from both experiments were conducted as shown in Figure 3 to make certain that there was no bias in relative quantitation, which is a typical quantitation mode in glycomics.

**Figure 4:**
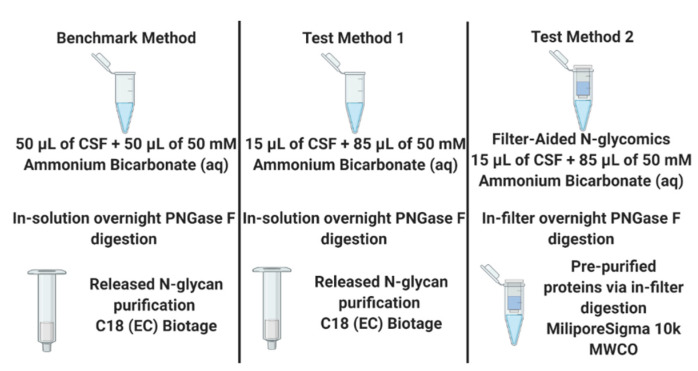
Workflows describing each method of releasing *N*-glycans from CSF as well as purification steps. The benchmark method was the method previously published by Cho et al., and the other two test methods were one in-solution digestion with 15 µL of CSF and one Filter-Aided *N*-Glycan Separation technique with 15 µL of CSF.

Even though the injection amount was decreased by half, the linear correlation of relative quantitation between the two different injection amounts suggested that CSF *N*-glycome profiling with an injection amount of 5 µL CSF was comparable to a 10 µL CSF injection. Since *N*-glycome profiling with an injection amount of 5 µL CSF can be accomplished as effectively as an injection amount of 10 µL CSF, two different sample preparation methods were investigated using the previously published method by Cho et al. as the benchmark, where a total of 50 µL of CSF was used to prepare the sample but only 10 µL of 50 µL CSF was injected. Figure 4 describes three different sample preparation methods tested in this work.

**Figure 5:**
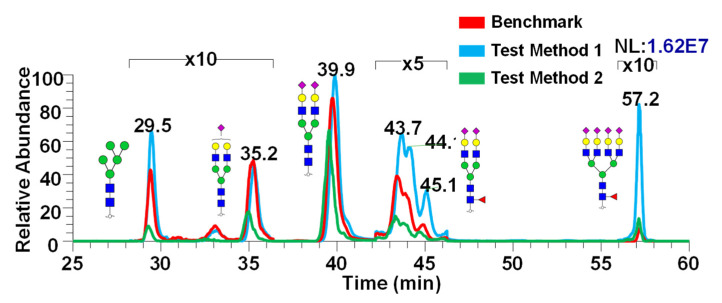
Representative chromatogram depicting the comparison between the three methods. Chromatograms are zoomed in accordingly to better display the peaks. Glycan cartoons are used as described in Figure 1. Glycan structures shown here are putative.

Figure 5 shows the representative chromatogram showing the differences in intensities between the three different methods exhibited in Figure 4. Differences in intensity show the effect of various sample preparation methods applied to release the glycans from CSF. It appears that Test Method 1 has demonstrated the highest intensities compared to the other two methods, which indicated its effectiveness in PNGase F digestion as well as the purification method.

**Figure 6:**
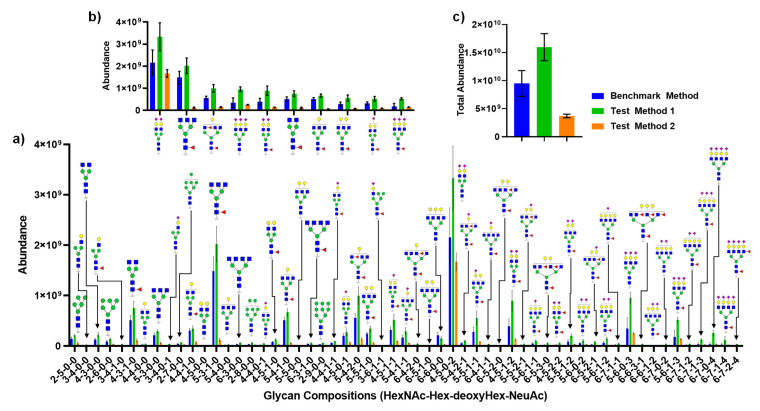
Quantitative analysis comparing three different methods of *N*-glycan release from CSF: (**a**) absolute abundance of all glycans detected; (**b**) absolute abundance of top ten most abundant glycans; (**c**) sum of total glycan abundance from each method. Glycan structures shown here are putative.

Figure 6 depicts the quantitative analysis of *N*-glycans released from CSF by each method described in Figure 4. Figure 6a compares individual glycans and their absolute abundances from two test methods against the benchmark method. Figure 6b describes the top ten most abundant glycans, while Figure 6c shows the sum of all glycan abundances. Interestingly, Test Method 2 (which utilized a 10k MWCO filter) showed a disproportional amount of decrease in abundance relative to the most abundant glycan, the biantennary disialylated glycan. The total abundance also showed a substantial increase in signal for Test Method 1 against the benchmark method and Test Method 2. 

## 3. Discussion

Unlike other biofluids such as serum or plasma, CSF is a unique biofluid due to its direct contact with the central nervous system. What is more particular about CSF in terms of proteome or glycome analyses is the fact that CSF has significantly less protein content than serum or plasma. As a result of this, previous glycomics studies of CSF have used substantial amounts of the material to overcome the lack of signal generated by the deficient amount of analyte. This brings a unique challenge especially in glycan analysis, because a lower amount of protein equates to a smaller number of glycoproteins and thus a lack of analyte: the glycans. The previous work by Cho et al. [10] used 50 µL of CSF to prepare the sample and injected one-fifth of the prepared sample. In this work, we attempted to reduce the starting material to 15 µL by applying either the same technique from Cho et al. or the Filter-Assisted *N*-Glycomics (FANGS) platform, which was adopted from Hecht et al. [36]. The latter was chosen to examine the possibility of avoiding the sample purification step with C18 cartridges after the *N*-glycan release by PNGase F and using 10k MWCO filters to pre-concentrate the proteins prior to the *N*-glycan release to facilitate the removal of the matrix. It should also be noted that although the use of C18 cartridges with graphitized carbon SPE is routinely performed in glycan analysis, graphitized carbon SPE steps were omitted in this workflow, since all three methods tested in this work have utilized online purification steps where trap column was used to desalt permethylated glycans prior to separation, which have been shown to enhance sensitivity compared to other purification techniques such as liquid–liquid extraction and offline C18 SPE [37]. 

To evaluate these techniques, we first compared a 10 µL injection from a 50 µL sample preparation and a 5 µL injection from the same sample. This was to examine whether the instrument could provide an adequate signal of glycans even though the injection amount had been halved. As shown in Figure 2, signal intensities were decreased by nearly half. This was an expected result, as half the amount of the sample was injected. However, this indicated that injecting 5 µL of the sample prepared from 50 µL is comparable to injecting 10 µL as previous work (benchmark) suggested, since the same number of glycans was detected in both cases. To further confirm this result, a scatter plot was drawn to investigate the relationship between the relative quantitation of the two injections, as shown in Figure 3. Since a linear relationship between the two methods was demonstrated, it was suggested that the two methods were comparable.

Since the 5 µL injection from the benchmark method provided a sufficient signal for an *N*-glycomics study of CSF, two different sample preparation methods were tested against the benchmark method with a 5 µL injection, as shown in Figure 4. Test Method 1 used our target CSF volume (15 µL) but increased the amount of 50 mM ammonium bicarbonate buffer to 85 µL to accommodate the same total digestion volume as the benchmark (100 μL). However, this increased the enzyme to substrate ratio in Test Method 1 compared to the benchmark, since the same amount of PNGase F was used to release the *N*-glycans. Test Method 2 involved FANGS, which incorporates 10,000 or 30,000 MWCO (Molecular Weight Cut-Off) regenerated cellulose filter units to pre-concentrate the proteins and remove the matrix prior to the enzymatic digestion. This methodology was chosen first in order to not only remove the unwanted biological matrix but also to remove salts that could not be removed with the C18 glycan purification step; second, since the matrix had been removed, further purification steps using C18 cartridges or porous graphitized carbon sorbents could be avoided, reducing the potential sample loss. 

Interestingly, Test Method 1, an in-solution digestion with a smaller enzyme to substrate ratio, showed high sensitivity versus both the benchmark and Test Method 2, as shown in Figure 6. It is possible that this result suggests that the increased amount of ammonium bicarbonate buffer solution may improve the digestion efficiency, which also indicates that the amount of buffer added during the benchmark method was not adequate to achieve an optimum PNGase F digestion condition. It is notable to mention that a total of 57 glycans were detected in this experiment, which is lower than the previous study where 72 glycans were detected using the same preparation technique [10] but with different mass spectrometers. However, there was no decrease in the number of glycans detected intra-experiment, which indicates that signals of minor glycan structures derived from CSF were already below the detection limit set by the detector. The number of glycans detected in this experiment is an improvement from the previous works from Fogli et al. [22], Goyallon et al. [32], and others [15,38,39,40] considering that the amount of glycans were derived from equivalent of 15 µL compared to 25 µL by Goyallon et al. [32] and 250 µL by Fogli et al. [22].

A more startling result was the fact that Test Method 2, where FANGS was utilized, not only demonstrated a lower amount of total glycan abundance but also showed a biased ratio of glycan abundances against the benchmark method, as shown in Figure 6b, where the top ten most abundant glycans were compared. This is a different observation made by Hecht et al., where there were no significant differences between FANGS and the carbograph SPE method. However, Zhu et al. [41] have compared in-solution PNGase F digestion against the FANGS using model glycoproteins where a distorted distribution of glycans was found using FANGS and determined that glycan distribution using the in-solution digestion method was more closely matched with NMR data. Moreover, the loss of sensitivity with FANGS determined by Zhu et al. agrees with the current study where the total signal loss was more than 50% with FANGS compared to the in-solution digestion. This result hints that perhaps a loss of proteins occurred during the purification step or the loss of the released glycans due to their potential bindings to the regenerated cellulose membrane, which resulted in a biased distribution of glycan abundances. 

Here, in this work, we demonstrated an improved method for profiling *N*-glycans of CSF. The method was compared against the benchmark method, which was previously published and against FANGs, an in-filter digestion method. We report that profiling of *N*-glycans in CSF using only 15 µL of CSF and injecting glycans derived from 5 µL of CSF showed higher sensitivity compared to the benchmark method as well as the FANGs technique, despite the fact that the protein concentration of CSF is considerably lower than those of other biofluids such as serum or plasma. 

## 4. Materials and Methods 

Iodomethane, sodium hydroxide beads, acetic acid, and ammonium borane complex were purchased from Sigma Aldrich (St. Louis, MO, USA). Isolute^®^ C18 (EC) cartridges were purchased from Biotage (Charlotte, NC, USA) and 10k Amicon Ultra-0.5 mL Centrifugal Filters were purchased from Millipore Sigma (Burlington, MA, USA). Microspin columns were purchased from Harvard Apparatus (Hollison, MA, USA). *N*-glycosidase F enzyme (PNGase F) was acquired from New England Biolabs (Ipswich, MA, USA). Solvents, including high-performance liquid chromatography (HPLC)-grade water, acetonitrile, methanol, and dimethyl sulfoxide were purchased from Fisher Scientific (Pittsburgh, PA, USA). Pooled CSF was acquired from Golden West Biologicals, Inc. (Temecula, CA, USA).

### 4.1. N-Glycans Release and SPE-C18 Purification

CSF samples of 15 and 50 μL were diluted to a total volume of 100 μL with 50 mM ammonium bicarbonate buffer (ABC buffer pH ≈ 7.5) and denatured in boiling water for 15 min. After the samples had cooled to room temperature, 1 μL of PNGase F (1000 U) was added, and the samples were incubated at 37 °C for 20 h. After incubation, the samples were dried using a Labconco CentriVap benchtop vacuum concentrator (Kansas City, MO).

Dried samples were resuspended with 300 μL of 5% acetic acid. The SPE-C18 cartridges were washed with 3 mL of methanol and then equilibrated with 3 mL of 5% acetic acid. Resuspended samples were applied to the SPE-C18 cartridges and washed with 300 μL of 5% acetic acid three times while all flow-through was collected and dried using the vacuum concentrator. 

### 4.2. N-Glycans Release and Purification using FANGS

The FANGS method was adopted from Hecht et al. with minor modifications. CSF samples of 15 μL were diluted to a total volume of 100 μL with ABC (Ammonium Bicarbonate) buffer and denatured in boiling water for 15 min. The denatured samples were loaded onto the previously washed filter membrane and equilibrated with 500 μL of water and 100 μL of ABC buffer, respectively. The samples were washed two times with 100 μL of ABC buffer and then resuspended in another 80 μL of the same buffer. One microliter of PNGase F (1000 U) was added to the sample solution and incubated at 37 °C for 20 h. After enzymatic digestion, the samples were eluted from the filter by centrifugation, the sample solution was recovered in a 1.5 mL tube, the filter was washed twice with 100 μL of ABC buffer, and the solution was recovered in the sample tube. The total collected sample was dried using the vacuum concentrator. 

### 4.3. Reduction of Glycan Reducing Ends and Permethylation

Digested and purified samples from SPE-C18 and FANGS protocols were reduced and permethylated according to the following procedure. The glycan reduction was accomplished by the addition of 10 μL of 10 μg/μL ammonium borane solution, which was followed by incubation at 60 °C for 1 h. After incubation, the residual borane was removed by the addition of methanol, generating methyl borate that was evaporated while drying in the vacuum concentrator.

Reduced *N*-glycans were subjected to solid-phase permethylation. Dried glycans were resuspended in 30 μL of dimethyl sulfoxide (DMSO), 1.2 μL of water, and 20 μL of iodomethane. The solution was applied into a microspin column packed with sodium hydroxide beads that were subsequently incubated in darkness at room temperature for 25 min. After the initial incubation period, 20 μL of iodomethane was applied to the spin column, and the reaction was allowed to procced for an additional 15 min. Permethylated *N*-glycans were dried and resuspended in aqueous mobile phase for LC-MS analysis.

### 4.4. LC-MS Analysis

A Dionex Ultimate 3000 nanoLC system (Thermo Scientific, San Jose, CA, USA) was coupled with an Exactive mass spectrometer (Thermo Scientific, San Jose, CA, USA). Permethylated glycans were first desalted with an online purification system using a C18 Acclaim PepMap 100 trapping column (2 cm, 75 μm internal diameter, 3 μm particle size, 100 Å pore size, Thermo Scientific). Purified permethylated *N*-glycans were separated using a C18 Acclaim PepMap column (15 cm, 75 μm internal diameter, 2 μm particle size, 100 Å pore size, Thermo Scientific). Samples were desalted using the online system with 3 μL/min of flow rate with 0.1% formic acid and 2% acetonitrile in water for 10 min. Mobile phase A consisted of 0.1% formic acid in water, while mobile phase B was 0.1% formic acid in acetonitrile. The chromatographic gradient was as follows: mobile phase B was held at 20% with 350 nL/min of flow rate for the first 10 min, at which point it was increased to 42%. Next, mobile phase B was increased from 42% to 55% from 10 to 50 min, then increased to 90% over 5 min, and finally reduced to 20% over 5 min. The mass spectrometer was set to full-scan mode, with a scan range from 700 to 2000 *m/z* and a mass tolerance within 10 ppm with the mass resolution set at 50,000.

### 4.5. Data Analysis

The analysis of the raw data was performed using Xcalibur 4.2 (Thermo Scientific) software; extracted ion chromatograms of each glycan structure, including all possible sodium and ammonium adducts, were generated. To profile *N*-glycans in the samples, relative quantitation was chosen to observe full profiles of *N*-glycans expressed in the CSF samples. The *m/z* values of target glycan structures, as well as their corresponding adducts, were applied to generate the extracted ion chromatograms (EICs). The areas under the curve of these EICs were integrated, and the generated data were used to perform a relative glycan quantitation. Statistical analysis was performed using GraphPad Prism.

## Data Availability

The data presented in this study are available on request from the corresponding author.

## Sample Availability

Samples of the compounds are not available from the authors.
